# Spinal Segmental Myoclonus in Primary Progressive Multiple Sclerosis

**DOI:** 10.5334/tohm.862

**Published:** 2024-02-26

**Authors:** Mariano Ruiz-Ortiz, Julián Benito-León

**Affiliations:** 1 Department of Neurology, University Hospital “12 de Octubre”, Madrid, Spain; 2 Instituto de Investigación Sanitaria Hospital 12 de Octubre (imas12), Madrid, Spain; 3 Centro de Investigación Biomédica en Red Sobre Enfermedades Neurodegenerativas (CIBERNED), Madrid, Spain; 4 Department of Medicine, Faculty of Medicine, Complutense University, Madrid, Spain

**Keywords:** Multiple Sclerosis, Movement Disorders, Spinal Myoclonus

## Abstract

**Background:**

A wide variety of associated movement disorders has been described in multiple sclerosis.

**Phenomenology Shown:**

A 57-year-old woman with primary progressive multiple sclerosis developed spinal segmental myoclonus associated with focal myelitis.

**Educational Value:**

Movement disorders in multiple sclerosis are phenomenologically diverse and have varied pathophysiological mechanisms, making it essential to identify them to initiate appropriate treatment.

## Background

While movement disorders in multiple sclerosis (MS) are not considered classic symptoms, a wide variety has been described. These include restless leg syndrome, tremor, ataxia, parkinsonism, and paroxysmal disorders such as ballismus, facial myokymias, hemifacial spasm, tic/tourettism, dyskinesias or myoclonus [1,2,3]. Authors herein show an infrequent finding: spinal segmental myoclonus associated with focal myelitis in a patient with primary progressive multiple sclerosis.

## Phenomenology Shown

**Figure 1:**
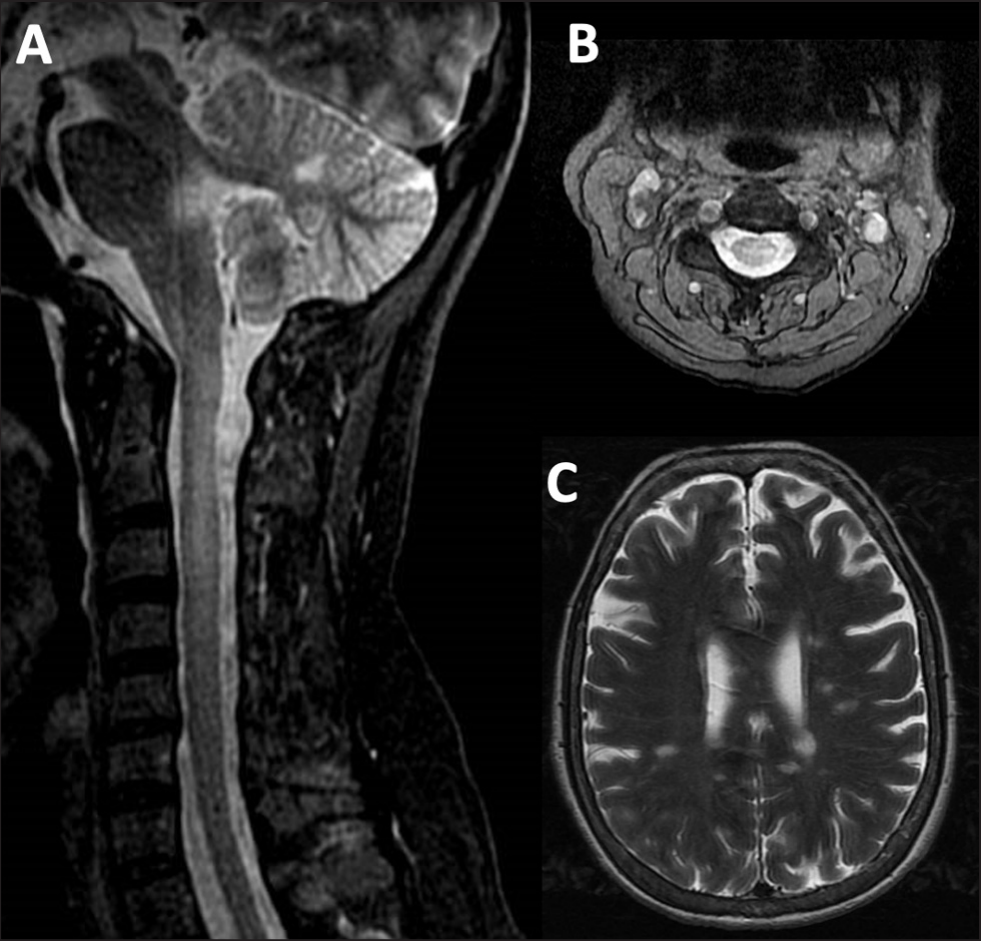
Sagittal **(A)** and axial **(B)** T2-weighted images with fat suppression highlight an area of myelitis at the C3–C4 level. Notably, hyperintensity is observed in the posterolateral region of the spinal cord. Additionally, axial T2-weighted imaging further reveals characteristic demyelinating lesions **(C)**.

A 57-year-old woman started two years earlier with progressive gait disturbance and cognitive complaints without any previous relapse. She had a history of smoking, non-cirrhotic portal hypertension (under evaluation), and anxious-depressive syndrome with an eating disorder. A neurological exam showed saccadic pursuit, mild proximal paraparesis, hypopalesthesia in lower limbs, and mild ataxic gait. Motor and sensory function in the upper limbs was preserved. Cranio-cervical magnetic resonance imaging (MRI) showed several typical demyelinating lesions involving juxtacortical, subcortical, periventricular, and infratentorial regions. Also, an area of myelitis was observed at C3–C4 (Figure 1) and D6–D7. Oligoclonal bands were positive. Applying the 2017 McDonald diagnostic criteria, a diagnosis of primary progressive MS was made. The patient did not start any disease-modifying treatment.

One year later, she developed brief but annoying spontaneous contractions of her left arm that worsened during periods of stress or increased activity. Video 1 showed those irregular myoclonic jerks involving the upper trunk and left arm. Needle electromyography showed bursts of normal muscle activity lasting 50 to 120 milliseconds. She was diagnosed with segmental spinal myoclonus associated with focal myelitis. She started levetiracetam 1000 mg daily with a partial response.

**Video 1:** **(Spinal Segmental Myoclonus in a Patient with Progressive MS):** This video captures a 57-year-old female exhibiting involuntary, arrhythmic, jerky movements localized to her upper trunk and proximal left arm, characteristic of spinal segmental myoclonus.

## Educational Value

Symptomatic oculo-palatine myoclonus in lesions of the Guillain-Mollaret triangle, intentional myoclonus due to lesions of the red nucleus, and segmental spinal myoclonus have been reported in MS [1].

Spinal myoclonus is characterized by involuntary, brief, semi-rhythmic contractions of muscles belonging to a spinal segment [4]. They are frequently precipitated by fatigue and stress and wane during sleep [4]. There are documented cases of spinal myoclonus due to demyelinating lesions within the cervical spinal cord, involving both the shoulder and arm as the initial manifestation of primary progressive MS, thus presenting a significant diagnostic challenge [3]. We feel that the segmental myoclonus of our patient was directly attributable to cervical cord demyelinating lesions.

The potential pathophysiological mechanisms underlying spinal myoclonus in MS are thought to involve axonal hyperexcitability and spontaneous neuronal discharge [1,2,3]. These phenomena may result in the disinhibition of alpha-motor neurons and the disruption of spinal interneuronal circuitry [1,2,3].

Managing spinal myoclonus involves using established antimyoclonic agents, including clonazepam, levetiracetam, valproic acid, or tizanidine, although the evidence supporting their effectiveness is limited [1,2,3].

In conclusion, although we do not usually focus on movement disorders when evaluating patients with MS, there is a wide range of them. They can be phenomenologically diverse and have varied pathophysiological mechanisms, requiring an appropriate diagnostic and therapeutic approach.
